# Data Resource Profile: EST-Health-30

**DOI:** 10.1093/ije/dyag113

**Published:** 2026-07-16

**Authors:** Sulev Reisberg, Marek Oja, Kerli Mooses, Sirli Tamm, Ami Sild, Harry-Anton Talvik, Sven Laur, Raivo Kolde, Jaak Vilo

**Affiliations:** Institute of Computer Science, University of Tartu, Tartu, 51009, Estonia; STACC, Tartu, 51009, Estonia; Institute of Computer Science, University of Tartu, Tartu, 51009, Estonia; Institute of Computer Science, University of Tartu, Tartu, 51009, Estonia; Institute of Computer Science, University of Tartu, Tartu, 51009, Estonia; Institute of Computer Science, University of Tartu, Tartu, 51009, Estonia; Institute of Computer Science, University of Tartu, Tartu, 51009, Estonia; STACC, Tartu, 51009, Estonia; Institute of Computer Science, University of Tartu, Tartu, 51009, Estonia; Institute of Computer Science, University of Tartu, Tartu, 51009, Estonia; Institute of Computer Science, University of Tartu, Tartu, 51009, Estonia; STACC, Tartu, 51009, Estonia

**Keywords:** Estonia, health dataset, EHR, pharmacy, linked data, OMOP

Key Features

- EST-Health-30 is a population-representative dataset of complete health records for a random 30% sample of the Estonian population (∼500 000 individuals) spanning 2012 to the present, enabling population-level epidemiological analyses with annual updates.
- The dataset is constructed by using a random sampling approach based on hashed password-protected personal identifiers, ensuring consistent inclusion over time with unbiased population coverage.
- Individual-level data are linked across multiple nationwide databases, including electronic health records, claims, prescriptions, and cancer and cause-of-death registry data, enabling multimodal analyses of health trajectories.
- All data are standardized to the Observational Medical Outcomes Partnership (OMOP) Common Data Model (CDM) version 5.4 using international vocabularies (e.g. SNOMED CT, RxNorm, LOINC), supporting reproducibility and participation in federated research networks.
- The dataset is accessible through a secure processing environment compliant with the European Health Data Space framework.

## Data resource basics

EST-Health-30 is a population-based health dataset that includes complete health records for a randomly sampled 30% of the Estonian population, linked across five national databases: electronic health records from primary and secondary care, claims, laboratory test results, pharmacy data, and cancer and cause-of-death registries, from 2012 onwards. The dataset currently contains data through to 2024 and includes 509 858 individuals.

It is updated annually through to 2026, with each release typically including data up to the end of the previous calendar year. The expected lag between data generation and availability is ∼6–24 months, depending on the source (e.g. manually curated cancer registry data may have longer delays). The dataset is fully mapped to the Observational Medical Outcomes Partnership (OMOP) Common Data Model (CDM), enabling a wide range of study designs.

## Data collected

### Source data

#### Source population

Estonia is a Northern European country on the Baltic Sea with a population of ∼1.37 million, of whom ∼70% are ethnic Estonians. Despite negative natural growth since the 1990s, the population has remained relatively stable since 2000 due to positive net migration, with a spike in 2022 driven by Ukrainian refugees (2.6% increase). The life expectancy for those born in 2024 is 79.5 years (75.0 years for men and 83.4 years for women), which is slightly below the European Union (EU) average of 81.7 years [1, 2].

#### Estonian healthcare system

A comprehensive overview of the Estonian healthcare system is provided by Kasekamp *et al.* [3]. Around 94% of the population is insured through the state-funded system managed by the Estonian Health Insurance Fund (EHIF). Roughly half of those insured are covered via employment-based or state contributions, while the remainder—such as children and individuals who are unable to work—receive coverage without contributing directly. EHIF finances a broad range of healthcare services and even those without insurance have access to emergency care, cancer screening, treatment for HIV and tuberculosis, and smoking or addiction cessation programs. Beyond the primary care system, which is based on family doctors, patients typically pay in full or share costs for dental services (except for children aged <19 years), long-term care, and many medications, particularly over-the-counter ones. In 2023, Estonia had the highest rate of self-reported unmet need for medical examination—i.e. needed consultations, diagnostic services, or treatment that were foregone—in the EU (12.9% vs EU average of 2.4%), driven primarily by waiting times [3]; consequently, many patients pay out of pocket for faster access to specialist care.

#### Source datasets

Estonia is a leader in delivering essential public services through digital platforms [4]. Regardless of whether individuals are insured, it is mandatory to collect and store health information in national databases for all individuals, resulting in longitudinal data. The EST-Health-30 dataset, compiled in 2025, integrates data from five national real-world health datasets in Estonia:

- The Estonian National Health Information System (ENHIS), operational since 2008, collects primary and secondary care summaries (including both in- and outpatient records), referrals, referral responses (including laboratory results), and immunization records, comprising both structured and unstructured data. All healthcare providers are legally required to submit patient case summaries to ENHIS, regardless of insurance coverage. EST-Health-30 includes most ENHIS document types but excludes medical image files (radiology reports are retained), dental records, and ambulance reports. Documents retrieved from ENHIS are referred to as electronic health records (EHRs).
- The Health Insurance Fund Database—the records of which we hereafter refer to as claims—contains information on diagnoses, procedures, claim costs, and insurance coverage periods, with data collected since the mid-1990s.
- The Estonian Medical Prescription Center, launched in 2010, contains information on prescribed and dispensed medications, excluding over-the-counter medications and drugs administered during hospital stays.
- The Cancer Registry, operational since 1968, records all cancer cases in Estonia. Due to manual curation, data are available with an ∼2-year delay.
- The Causes of Death Register contains the information on all deaths in Estonia since 1986 [5].

An overview of the document types included in the EST-Health-30 datasets is given in Table 1.

**Table 1:** Summary statistics of the source documents in the EST-Health-30 dataset.

Data source	Document type	Content examples	Document count	Patients with at least one document (*n*)	Mean document count per patient (total cohort)
ENHIS (EHR)	Outpatient care	Visits, diagnoses (ICD-10), anamnesis, objective findings, allergies, lab/pathology/endoscopy/radiology examination results (reports included, image files excluded), measurements, test results, procedures, surgeries, summary, health behaviour data in free-text parts	17 424 410	487 469	34.2
	Referral response	Results of lab tests, radiology examinations (reports included, image files excluded)	15 521 887	477 256	30.4
	Immunization	Immunization date and info	2 132 845	349 446	4.2
	Inpatient care	Same as “Outpatient care,” length of hospital stay, drugs administered during the stay (inconsistently recorded)	793 431	265 323	1.6
	Day care	Same as “Outpatient care”	243 349	125 350	0.48
	Home nursing	Visits, diagnoses (ICD-10), health behaviour data in free-text parts, overview of health status	8141	5326	0.016
	Inpatient nursing	Same as “Outpatient care”	2957	2314	0.006
The Health Insurance Fund Database	Claims: outpatient care	Visits, diagnoses (ICD-10), healthcare services provided, costs for the patient (for prescriptions only) and insurance fund	29 143 369	488 114	57.2
	Claims: dental care	Same as “Claims: outpatient care”	1 747 102	165 580	3.4
	Claims: inpatient care	Same as “Claims: outpatient care”	784 614	263 840	1.5
	Claims: nursing care	Same as “Claims: outpatient care”	330 627	45 301	0.65
	Claims: day care	Same as “Claims: outpatient care”	316 118	150 234	0.62
	Insurance period	Dates and the basis of the insurance period	3 549 258	496 620	7.0
Estonian Medical Prescription Center	Prescription	Prescription date, diagnosis (ICD-10), prescribed ingredients (ATC) and amount, dispensed package and amount, dispense date, costs for the patient and insurance fund	40 028 909	467 858	78.5
Cancer Registry	Cancer registry record (cancer case)	Tumour diagnosis, topography, morphology, TNM classification, date, general info on first-line treatment	31 241	28 569	0.061
Causes of Death Register	Death record	Death date, cause	61 822	61 822	0.12

“Document count” refers to the number of source documents (e.g. Health Level Seven Clinical Document Architecture documents or claims entries) rather than unique clinical encounters. “Mean document count per patient” reflects the average number of documents per individual accumulated over the full observation period (2012–24).

ATC, Anatomical Therapeutic Chemical classification system; ICD-10, International Classification of Diseases, 10th Revision.

### Data-collection method

In the EST-Health-30 dataset, information from national health databases is linked at the individual level by using a unique personal ID—an immutable 11-digit identifier assigned to all Estonian citizens at birth and to permanent residents upon arrival. This identifier enables deterministic linkage across databases. The current dataset includes records from 2012 to 2024, with updates planned through 2026.

### Sampling

EST-Health-30 contains complete health records for a randomly selected 30% sample of individuals appearing in the national databases. To generate this cohort, all data holders applied a shared SHA-256 hashing procedure to personal IDs padded with a secret key. Individuals whose hash values met predefined criteria—based on specific symbol positions—were included, ensuring exactly 30% random selection. The resulting SHA-256 digests serve as pseudonyms that uniquely identify individuals within the dataset. Because hashing is applied only to personal identifiers already present in the contributing databases, the dataset includes exclusively individuals with at least one record in at least one source; residents with no health-system footprint are not represented and do not enter the selection process.

For privacy protection, the only private information exchanged between database owners was a common secret key, used for hashing, as each party had knowledge of their own personal IDs already. No raw data were shared and the secret key remained unknown to the EST-Health-30 research team, preventing re-identification. The procedure allows data owners to process their data independently while allowing the data recipient to link records by the same pseudonym (hash value), accounting for newly added individuals and maintaining consistent randomization over time.

**Figure 1:**
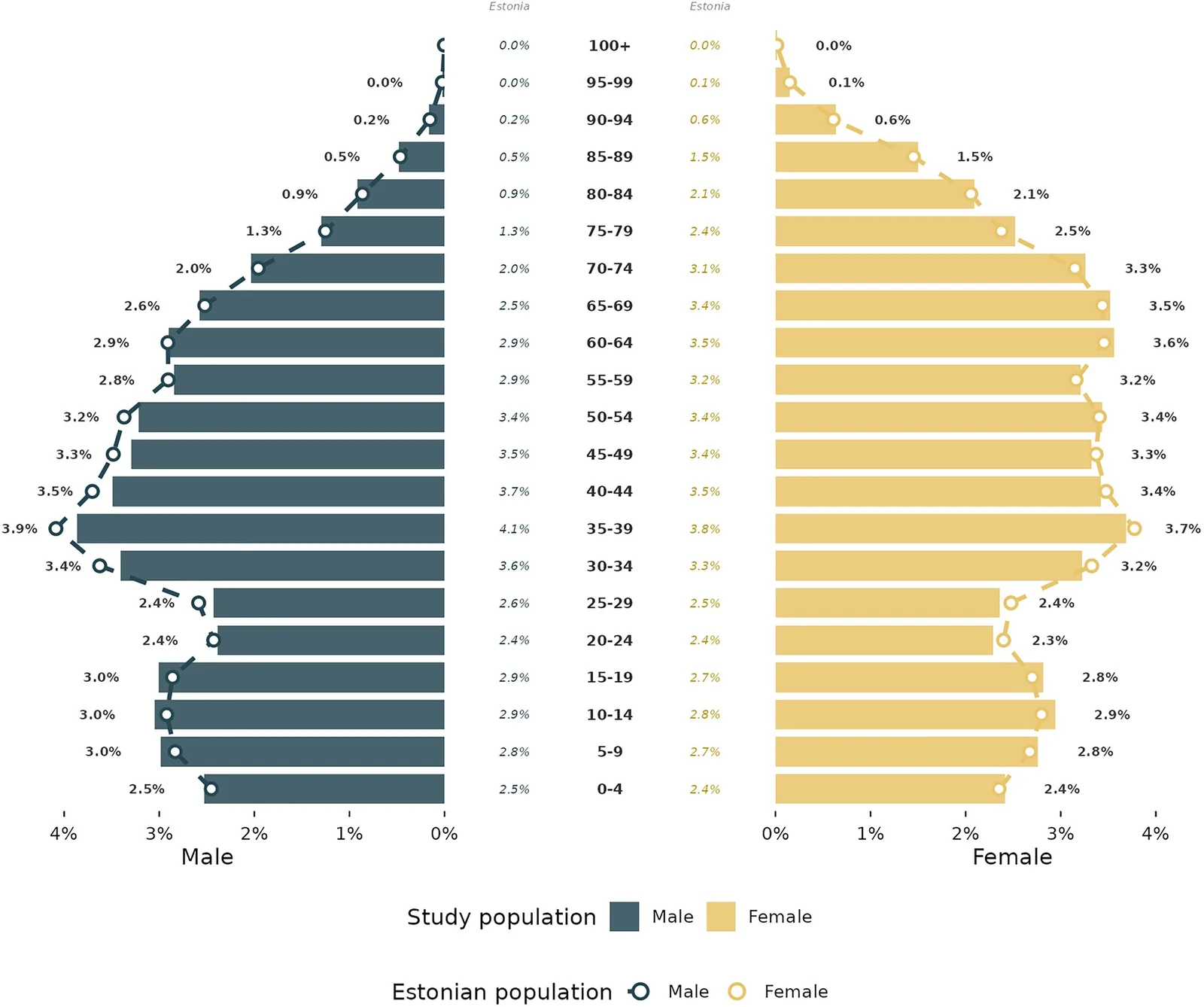
Population pyramid of the EST-Health-30 dataset (*n* = 396 743) compared with official Estonian population (*n* = 1 374 687) on 1 January 2024.

Figure 1 gives an overview of the population of the EST-Health-30 dataset compared with the official Estonian population on 1 January 2024. In total, the resulting database contains health data of 509 858 individuals (244 661 males, 47.99%, and 265 095 females, 51.99%, and 102 with unspecified gender, 0.02%). Of these, 396 743 had an active observation period on 1 January 2024 (i.e. were under observation on that date).

### Data harmonization

All data sources are mapped to the OMOP CDM version 5.4 and its standard vocabularies by using the transformation pipeline described by Oja *et al.* [6] and Talvik *et al.* [7]. This includes transforming original International Classification of Diseases, 10th Revision (ICD-10) diagnosis codes and medication package codes to SNOMED CT and RxNorm. Source codes are retained alongside standardized concepts, allowing analyses to be performed via either representation, depending on the use case. Using the rule-based extraction process described by Talvik *et al.* [7], the following variables were also extracted from free-text fields written in Estonian and mapped to OMOP: body height, weight, body mass index, waist circumference, blood pressure, total-, low-density lipoprotein (LDL), and high-density lipoprotein (HDL) cholesterol, estimated glomerular filtration rate, left ventricular ejection fraction, prostate-specific antigen, Eastern Cooperative Oncology Group performance status, and tobacco-smoking status. Free-text parts from the EHRs are stored in the notes table in the OMOP CDM.

Table 2 provides an overview of the main OMOP domains (tables) and their record counts in the resulting dataset. As the dataset does not include migration records, observation periods were derived from available healthcare contacts and insurance coverage periods, and constrained by birth, death, and dataset boundaries (2012–24). For a small number of individuals with insufficient information to define a meaningful observation period, a sentinel value was assigned to allow identification and exclusion in analyses where appropriate. The lower mapping rate observed in the procedure domain (84.3%) primarily reflects limitations of local procedure coding systems rather than deficiencies in data quality. Many local procedure codes are broad or ambiguous, covering multiple distinct procedures under a single code, and therefore do not have direct equivalents in standard vocabularies and require ongoing mapping efforts. **Supplementary Table S1** presents the 10 most prevalent concepts within each OMOP domain.

**Table 2:** Main domains (tables) and their features in the EST-Health-30 OMOP dataset.

Domain	Type of available information	Record count	Person count	Percentage of persons	Classifiers used (source and OMOP standard vocabulary)	Distinct source code count	Number of distinct source codes mapped to OMOP standard vocabularies	Percentage of total codes mapped	Percentage of total records mapped
Measurement	Date, value, unit, range	248 358 785	493 664	96.8	LOINC, Estonian local healthcare service codes, Cancer Modifier, OMOP Extension, SNOMED CT, ICD-10, SNOMED, NCSP	5874	4776	81.3	97.4
Cost	Total, paid by person and paid by insurance cost records for all domains	166 179 255	494 711	97.3	Euros	NA	NA	NA	NA
Note	Texts from EHR (in Estonian)	112 812 550	495 880	97.3	NA	NA	NA	NA	NA
Condition	Start and end date of the condition (mostly diagnosis), cause of death (if applicable)	109 820 859	493 044	96.7	SNOMED CT, ICD-10, ICD-O-3, OMOP Extension, NCSP, Estonian local healthcare service codes	16 305	16 073	98.6	100.0
Document (visit in OMOP)	Document type, date or case dates, care site	108 477 761	509 851	100.0	NA	20	20	100.0	100.0
Observation	Date, value, and unit or qualifier	86 673 047	493 504	96.8	SNOMED CT, DRG, LOINC, OMOP Extension, Estonian local healthcare service codes, ICD-10, NCSP	14 163	12 230	86.4	97.7
Drug	Date of prescription and dispense, ingredient, package code, quantity, days’ supply	41 992 793	476 413	93.4	RxNorm, ATC, Estonian local drug package codes, CVX, Estonian local healthcare service codes, NCSP, SNOMED CT	5511	5156	93.6	99.3
Procedure	Date and procedure	36 815 875	488 349	95.8	SNOMED CT, Estonian local healthcare service codes, ICD-10, NCSP	8088	4338	53.6	84.3
Person	Birth year, gender, death date	509 858	509 858	100.0	NA	NA	NA	NA	NA
Observation period	Start and end date of the observation period of the person	509 858	509 858	100.0	NA	NA	NA	NA	NA

SNOMED CT, Systematized Nomenclature of Medicine Clinical Terms; LOINC, Logical Observation Identifiers Names and Codes; ATC, Anatomical Therapeutic Chemical classification system; NCSP, Nomesco Classification of Surgical Procedures; ICD-O-3, The International Classification of Diseases for Oncology, 3rd Edition; DRG, Estonian diagnosis-related groups; CVX, Vaccine Administered code set.

### Data quality

Data cleaning, standardization, and quality assessment were performed as part of the OMOP transformation pipeline, including validation, deduplication, and consistency checks, as described in Oja *et al.* [6] Standardized data-quality metrics were evaluated across all source datasets and OMOP domains to ensure completeness, consistency, and plausibility prior to release. This approach is aligned with the European Medicines Agency (EMA) Data Quality Framework [8] for real-world data, which emphasizes structured metrics, transparency of data provenance, and assessment of fitness for use in relation to a research question.

We additionally used the CdmOnboarding package [9] to generate an onboarding report of the type used by the DARWIN EU^®^ Coordination Centre and EMA to assess CDM readiness for regulatory studies. The report provides a comprehensive overview of all domains, including fill rates and mapping completeness. We also ran the DataQualityDashboard package [10], the 1625 applicable checks of which 1612 passed (99.2%). The 13 failures were reviewed manually and reflected expected data characteristics rather than quality problems.

To assess population representativeness, we compared the age–sex distribution of individuals alive on 1 January 2024 with official Statistics Estonia population figures for the same date (Fig. 1). The population size in EST-Health-30 is slightly below what would be expected from official Estonian population statistics (28.9% rather than 30.0%), particularly in the age group of 20–34 years. We attribute this primarily to recent immigrants who have no health insurance and have had no contact with the Estonian healthcare system.

This study follows the REporting of studies Conducted using Observational Routinely-collected Data (RECORD) statement [11]. A completed RECORD checklist is provided as **Supplementary Table S2**.

### Secure processing environment

All data processing is carried out in the Secure Analysis and Processing Unit (SAPU)—a secure processing environment hosted at the High Performance Computing Centre of the University of Tartu [12], which is fully compliant with the General Data Protection Regulation and European Health Data Space cybersecurity standards. These environments enable authorized researchers to process pseudonymized or anonymized health data without leaving the controlled infrastructure. SAPU operates in a tightly firewalled environment with no general internet connectivity. Researchers access the environment remotely through a browser-based virtual desktop. The data themselves never leave the secure perimeter and only aggregated results may be exported through a controlled disclosure procedure. All user activity is logged and recorded via continuous screen capture. In addition, SAPU is equipped with graphics processing unit infrastructure, enabling the use of transformer-based models, including large language models such as Google MedGemma [13], within the SAPU environment.

## Data resource use

EST-Health-30 supports a wide range of real-world evidence studies through its population coverage, longitudinal depth, and standardized OMOP structure. A key capability is the construction of detailed cohorts based on multimodal patient data.

For example, a heart failure cohort can be defined by combining diagnostic information, prescriptions, and laboratory measurements. Patients without prior heart failure diagnoses or related medication use can be identified at first presentation by using indicators such as elevated N-terminal pro-B-type natriuretic peptide (NT-proBNP, ≥125 ng/L) together with reduced ejection fraction (<40%). This illustrates the ability of the dataset to define clinically meaningful cohorts by using structured variables across multiple data domains.

**Figure 2:**
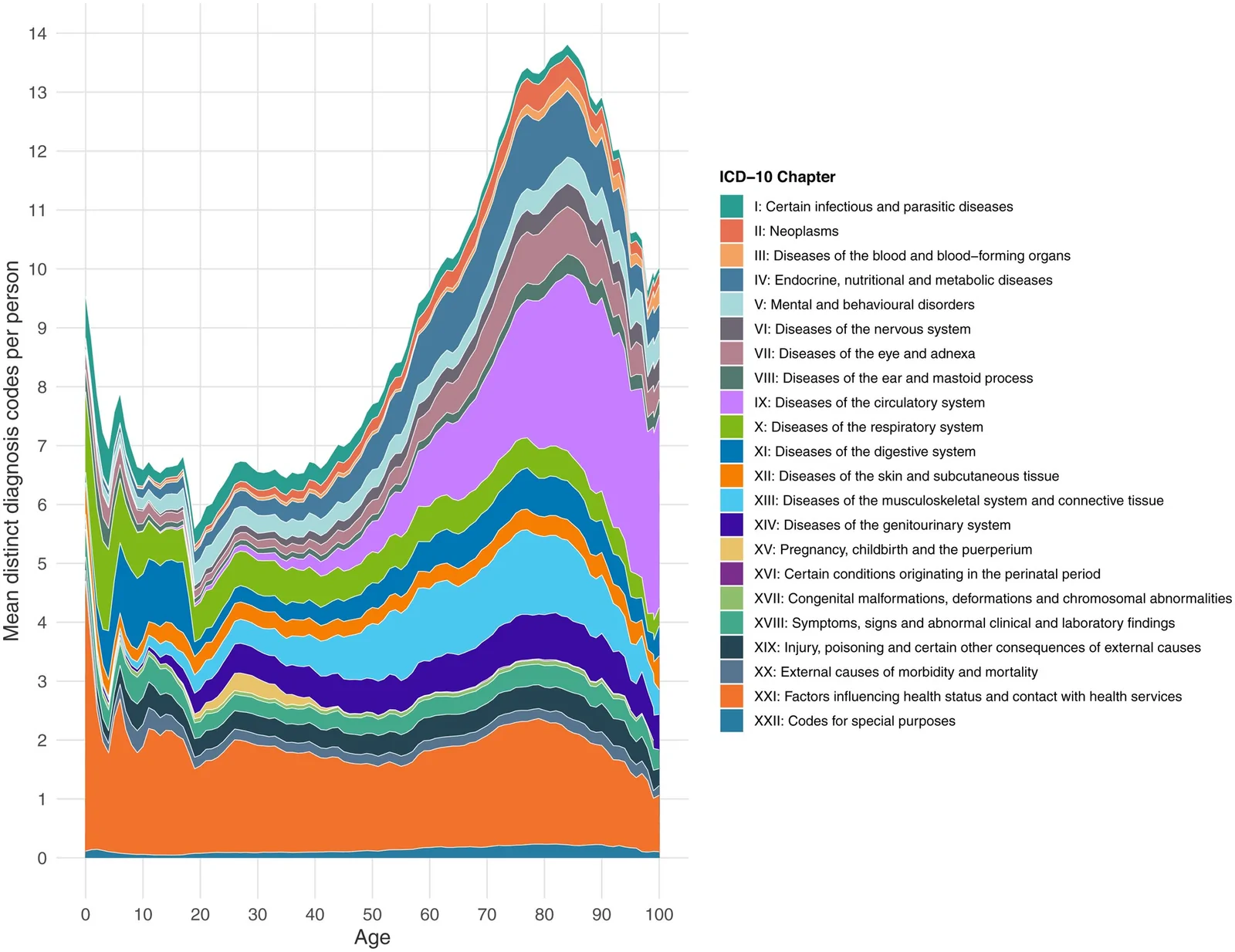
Age-specific distribution of diagnoses in 2024 by ICD-10 chapter. Values represent the mean number of distinct diagnoses per person at each age within each chapter. Estimates are smoothed by using a 2-year rolling window.

At the population level, the dataset enables the characterization of disease burden across the life course. Figure 2 presents age-specific counts of distinct diagnoses per person stratified by ICD-10 chapters (used here for descriptive aggregation), demonstrating the shift from respiratory conditions in early life to chronic cardiovascular, musculoskeletal, and metabolic diseases at older ages. A noticeable decline in diagnoses from the digestive system category in early adulthood reflects the transition at age 19 years when dental care is no longer routinely covered by the health insurance system, leading to fewer recorded diagnoses in this domain. These patterns illustrate the ability of the dataset to capture population-level morbidity and its evolution over time, while also highlighting how healthcare system characteristics influence the observed data.

The longitudinal structure supports studies of disease trajectories, healthcare utilization and costs, medication adherence, comparative effectiveness and safety (by using established causal inference methods), and the development and validation of predictive models for outcomes such as disease onset, hospitalization, or mortality. The dataset also supports methodological research on standardization, phenotyping, and reproducible analytics. Finally, OMOP mapping enables participation in international federated studies, in which only aggregated results are exchanged while preserving data privacy.

## Strengths and weaknesses

EST-Health-30 covers a random 30% of the Estonian population with demographic distributions closely aligned with national statistics, supporting generalizability and reducing selection bias. Deterministic linkage across five nationwide databases enables the comprehensive longitudinal capture of healthcare trajectories across care settings over more than a decade. The dataset provides rich multimodal clinical information—diagnoses, laboratory measurements, procedures, medications, and healthcare utilization—standardized to the OMOP CDM by using international vocabularies, enhancing interoperability and supporting federated research.

As with all routinely collected health data, the source data were not originally created for research purposes and may therefore have been subject to misclassification, incomplete recording, and unmeasured confounding. The dataset does not capture periods during which individuals may have resided outside Estonia, which may have led to incomplete follow-up for some individuals. Although EHRs are included, claims and costs for services fully paid out of pocket or provided in private settings are not recorded. Dentistry (except for claims data for children), medical image files, and in-hospital medication use are also not captured.

Some important clinical information is recorded only in free-text fields and may not yet be fully available for analysis, as extraction of these data is ongoing. Recovery of the original person identifiers is not possible for data users, as the pseudonymization key is held only by the original data providers. Although individual re-identification cannot be entirely excluded for any rich health dataset, the secure analysis environment and controlled disclosure procedures are designed to mitigate this residual risk. Caution is also required when combining EST-Health-30 with other Estonian datasets due to potential patient overlap. In addition, relationships between individuals are not recorded, limiting family-level analyses.

The dataset currently contains limited direct information on socioeconomic factors such as education, income, or occupation. Although proxy indicators, such as insurance status and healthcare utilization patterns, may be used, the lack of comprehensive socioeconomic data should be considered when addressing confounding in epidemiological studies.

Finally, despite including nearly 510 000 individuals, the dataset may still have limited statistical power for analyses of rare outcomes.

## Data resource access

EST-Health-30 was developed in accordance with the FAIR (Findable, Accessible, Interoperable, Reusable) data principles (https://www.go-fair.org/fair-principles/). The dataset will be made findable upon publication of this manuscript. Access is governed through a multi-step process requiring a cooperation agreement with the Health Informatics Research Group at the University of Tartu, ethics approval from the national scientific ethics committee coordinated by the Estonian Research Council, and subsequent authorization from the Ministry of Social Affairs. Following approval, a minimal dataset is provided via SAPU, from which only aggregated results may be exported. The process from study protocol submission to data access typically takes ∼4–5 months. Interoperability is achieved through standardization to the OMOP CDM, enabling the reuse of analytical tools, phenotype libraries, and federated network study protocols developed within the Observational Health Data Sciences and Informatics (OHDSI) community. Reusability is supported by comprehensive metadata, a detailed data dictionary, and harmonization documentation, as described in Oja *et al.* [6].

## Supplementary Material

dyag113_Supplementary_Data
